# An Amplicon-Based Application for the Whole-Genome Sequencing of GI-19 Lineage Infectious Bronchitis Virus Directly from Clinical Samples

**DOI:** 10.3390/v16040515

**Published:** 2024-03-27

**Authors:** Hoang Duc Le, Tuyet Ngan Thai, Jae-Kyeom Kim, Hye-Soon Song, Moon Her, Xuan Thach Tran, Ji-Ye Kim, Hye-Ryoung Kim

**Affiliations:** 1 Avian Disease Division, Animal and Plant Quarantine Agency, Gimcheon 39660, Gyeongsangbuk-do, Republic of Korea; lh.duc@ibt.ac.vn (H.D.L.); ttn267@korea.kr (T.N.T.); jaekum42@korea.kr (J.-K.K.); hssong1217@korea.kr (H.-S.S.); herm@korea.kr (M.H.); 2 Institute of Biotechnology, Vietnam Academy of Science and Technology, Cau Giay, Hanoi 11300, Vietnam; tranthach90@gmail.com

**Keywords:** infectious bronchitis virus, avian coronavirus, GI-19 lineage, whole-genome sequencing, amplicon-based genome sequencing, next-generation sequencing

## Abstract

Infectious bronchitis virus (IBV) causes a highly contagious respiratory disease in chickens, leading to significant economic losses in the poultry industry worldwide. IBV exhibits a high mutation rate, resulting in the continuous emergence of new variants and strains. A complete genome analysis of IBV is crucial for understanding its characteristics. However, it is challenging to obtain whole-genome sequences from IBV-infected clinical samples due to the low abundance of IBV relative to the host genome. Here, we present a novel approach employing next-generation sequencing (NGS) to directly sequence the complete genome of IBV. Through in silico analysis, six primer pairs were designed to match various genotypes, including the GI-19 lineage of IBV. The primer sets successfully amplified six overlapping fragments by long-range PCR and the size of the amplicons ranged from 3.7 to 6.4 kb, resulting in full coverage of the IBV genome. Furthermore, utilizing Illumina sequencing, we obtained the complete genome sequences of two strains belonging to the GI-19 lineage (QX genotype) from clinical samples, with 100% coverage rates, over 1000 × mean depth coverage, and a high percentage of mapped reads to the reference genomes (96.63% and 97.66%). The reported method significantly improves the whole-genome sequencing of IBVs from clinical samples; thus, it can improve understanding of the epidemiology and evolution of IBVs.

## 1. Introduction

The infectious bronchitis virus (IBV) is the primary cause of infectious bronchitis (IB), an acute and contagious disease in chickens. The rapid transmission and frequent occurrence of the disease have led to huge economic losses for the poultry industry worldwide [1]. IBV belongs to the genus *Gammacoronavirus*, family *Coronaviridae*, which is characterized by viruses possessing a positive single-stranded RNA genome. The IBV genome has a length of approximately 27.6 kilobases (kb) and comprises 5′ and 3′ untranslated regions and about 10 open reading frames (ORFs) coding for 4 structural proteins (spike glycoprotein (S), envelope protein (E), membrane protein (M), and nucleocapsid protein (N)) and other non-structural proteins [2,3]. The S glycoprotein is cleaved into subunits S1 and S2. The S1 subunit is responsible for host cell attachment and contains a receptor binding domain and hypervariable regions (HVR) [4,5,6]. Thus, the S1 gene has been widely used to classify IBV genotypes and serotypes [7]. Mutations caused by the high error rates of the viral RNA polymerase and recombination within the S1 unit have led to various strains or genotypes of IBV worldwide [2].

According to the most recent classification based on the S1 gene [7], IBVs can be divided into 6 genotypes (GI–GVI) and 32 distinct lineages. More genotypes (GVII–GIX) have been found in China and Mexico [8]. Most IBV genotypes are specific to a geographic region, and some countries have domestic lineages. In particular, the GI-19 lineage, known as the QX type, is one of the major genotypes worldwide and is the dominant lineage in Korea [9,10,11]. In 1991, nephropathogenic IBV of the KM91 type belonging to the GI-19 lineage emerged [12]. Since then, the QX type of the GI-19 lineage has predominated, and the K40/09 type, a recombinant of the KM91-type and QX-type strains, emerged in 2005 [13]. Previous studies have demonstrated that QX-type IBVs have recombined with various field strains, including vaccine strains, resulting in the emergence of novel variants and changes in the antigenic properties of IBV [14,15,16,17]. These mutations and recombinations continually lead to the emergence of new IBV strains, making the virus extremely difficult to identify and characterize [16,18].

Recently, it was reported that not only the S1 gene but also non-structural proteins may play critical roles in the replication and pathogenicity of IBV [19,20], suggesting that whole-genome sequencing is required to fully characterize IBV viruses and understand their epidemiology, including their antigenicity, tissue tropism, and pathogenicity [21]. Current advances in metagenomic next-generation sequencing (mNGS) technologies combined with Sequence-Independent, Single-Primer Amplification (SISPA) have provided a useful tool for uncovering the entire genome of IBV [22,23,24,25]. Generally, these methods have been applied to IBV isolates amplified by serial passage in embryonated chicken eggs [26,27,28]. However, despite the need for the mNGS of viruses in clinical tissue samples, variations in amplification due to the low viral genetic load in tissue samples can pose challenges for metagenomic sequencing [29,30]. To circumvent this difficulty, different enrichment techniques employing specific primers targeting the whole genome of the virus have been developed. Specific amplicon-based sequencing approaches have proven successful since they can generate sufficient amounts of genetic material for whole-genome sequencing [22,31].

In this study, we developed an amplicon-based sequencing method for the direct sequencing of the entire genome of IBV in clinical samples. Additionally, we evaluated whether the method was performant when used with the non-targeted SISPA approach with Illumina sequencing technology.

## 2. Materials and Methods

### 2.1. Virus Isolation and Propagation

The clinical samples, designated as AD04 and AQ10, were collected from the cecal tonsils of 23-day-old and 29-day-old broiler chicken carcasses, respectively, which were suspected of harboring IBV in South Korea in 2023. The chicken carcasses from two different farms were submitted for IBV diagnostic work-up at the Avian Disease Division, Animal and Plant Quarantine Agency. The chickens displayed evident respiratory symptoms and depression before death. Subsequently, IBVs were isolated from the clinical samples by injecting 0.2 mL of 10% tissue homogenates into the allantoic cavity of 10-day-old specific pathogen-free chicken embryonated eggs and incubating them at 37 °C for 72 h. The allantoic fluid from the inoculated eggs was collected for RNA extraction and stored at −70 °C for further use. The isolated samples were designated as AD04-CE1 and AQ10-CE1, respectively.

### 2.2. RNA Extraction and IBV Real-Time RT-PCR Assay 

The total RNA was extracted from the clinical samples and allantoic fluid using the TANBead Nucleic Acid Extraction Kit on an automated TANBead Maelstrom™ 4800 (Taiwan Advanced Nanotech Inc., Taoyuan, Taiwan) according to the manufacturer’s instructions. Additionally, the genomic DNA was removed from the extracted RNA using a DNA-free™ Kit (Thermo Fisher Scientific, Waltham, MA, USA) according to the manufacturer’s guidelines.

The IBV detection and quantification in the RNA samples were performed using real-time RT-PCR targeting the 5′-UTR region of IBV, as previously described [32]. The reaction mixture consisted of 10 μL RealMOD^TM^ Probe M^2^ 2X qRT-PCR mix (iNtRON Biotechnology Inc., Gyeonggi, Republic of Korea), 0.5 μL forward primer, 0.5 μL reverse primer, 0.5 μL probe, 3 μL RNA, and nuclease-free water in a final volume of 20 μL. Real-time RT-PCR was performed on a LightCycler® 96 Instrument (Roche, Basel, Switzerland) under the following conditions: reverse transcription at 50 °C for 10 min and initial denaturation at 95 °C for 10 min, followed by 40 cycles of 95 °C for 15 s and 60 °C for 1 min. The fluorescence intensities were acquired during the 60 °C step of each cycle, and the cycle threshold (Ct) value was analyzed using the LightCycler® 96 SW 1.1 software.

### 2.3. In Silico Design of the Primer Sets

Full-length sequences of IBV (n = 55) available in the National Center for Biotechnology Information (NCBI) GenBank (up until November 2023) were downloaded to design the universal primers (Table S1). The alignment of the genome sequences was performed to evaluate the nucleotide conservation across the IBV genomes with CLC Main Workbench software version 20.0.4 (Qiagen, Hilden, Germany) using a very accurate alignment algorithm. Six universal primer sets covering the entire genome (Table 1) were designed from conservative regions of the IBV genomes. The criteria for the primers were as follows: amplicon length, 3.7–6.4 kb; primer length, 19–24 nucleotides; number of degenerate bases < 4; and overlaps between amplicons > 140 bp. The primers were synthesized by Macrogen Inc. (Seoul, South Korea). The stock concentration was 100 pmol/µL, and working primer concentrations of 10 pmol/µL were obtained by diluting the stock solution in nuclease-free water (Table 1).

### 2.4. cDNA Synthesis and Amplicon Analysis

For the cDNA synthesis, 5 μL total RNA from each sample, together with an IBV gene-specific primer (5′-TACCGTTCGTTTCCA-3′), was subjected to reverse transcription using SuperScript IV Reverse Transcriptase (Thermo Fisher Scientific, Waltham, MA, USA) according to the manufacturer’s guidelines. Six individual amplicons were amplified using the following mixture: 10 μL LongAmp® Taq 2X Master Mix (New England Biolabs, Ipswich, MA, USA), 0.5 μL forward primer (10 pmol/µL), 0.5 μL reverse primer (10 pmol/µL), and 2 µL of cDNA in a final volume of 20 µL. The cycling conditions were as follows: 95 °C for 5 s, followed by 35 cycles of 95 °C for 45 s, 53 °C for 45 s, and 65 °C for 5 min, with a final extension at 65 °C for 10 min. The PCR products were run on a 1% agarose gel to confirm the presence of amplicons. The amplicons generated for each primer pair were pooled (in equal volumes) into a single mixture for library preparation and Illumina sequencing. The pooled mixture was purified/cleaned up using AMPure XP beads (Beckman Coulter, Brea, CA, USA) following the manufacturer’s instructions.

### 2.5. SISPA

The total RNA extracted from the clinical samples and isolated samples was used for SISPA, as previously described [25]. The dsDNA generated by SISPA was quantified using the Qubit dsDNA HS assay (Thermo Fisher Scientific, Waltham, MA, USA) according to the manufacturer’s guidelines.

### 2.6. Amplicon and Metagenome Sequencing Libraries Preparation

Libraries were prepared using the NEXTFLEX® rapid XP DNA-seq 2.0 kit for Illumina platforms (PerqkinElmer, Waltham, MA, USA). The quantification of the libraries was carried out using the Qubit dsDNA HS assay kit (Thermo Fisher Scientific, Waltham, MA, USA), and the size of the libraries was measured using the Agilent TapeStation 4200 (Agilent Technologies, Santa Clara, CA, USA). High-quality libraries were pooled to achieve equimolar concentrations, 1% PhiX Control v3 Library (Illumina, San Diego, CA, USA) was added, and then next-generation paired-end sequencing (2 × 150 bp) was performed on an Illumina MiniSeq instrument using the 300-cycle MiniSeq Mid Output Reagent Cartridge (Illumina, San Diego, CA, USA). 

### 2.7. Bioinformatic Analysis

The quality of the raw Illumina sequencing reads was assessed by fastp [33]. Adapter trimming was performed using Cutadapt and Trimmomatic with the default parameters [34]. The filtered reads were then aligned to the reference genome (IBV strain YX10, NCBI accession no. JX840411) using BWA-MEM [35] with the default settings. Subsequently, the mapped reads were retrieved using the Bamtofastq tool and subjected to de novo assembly using MEGAHIT software (v. 1.2.9) [36]. To evaluate the quality of the assembly, the Quality Assessment Tool for Genome Assembly (QUAST, v.5.0.2) was employed to compare the relative quality of each strain. The average coverage was calculated using Qualimap (v. 2.2.1) while examining the mapping of the raw sequence reads to the reference genome using BWA-MEM and Samtools.

For the genome annotation, the GATU program was utilized with the reference genome of the YX10 strain (accession no. JX840411) [37]. The pairwise identities were determined using CLC Main Workbench software version 20.0.4 (Qiagen, Hilden, Germany). 

### 2.8. Phylogenetic Analysis and Sanger Sequencing

The alignments of both the full genome and the S1 gene were conducted utilizing prototype strains from various lineages and reference strains. These alignments were executed using MAFFT version 7.467 software [38]. Phylogenetic trees were then generated by the neighbor-joining method, with 1000 bootstrap replications, using MEGA 11 software [39]. The nucleotide differences between the clinical and isolated samples based on NGS were verified by custom Sanger sequencing (Cosmo Genetech, Seoul, Republic of Korea).

## 3. Results

### 3.1. RT-qPCR and Generation of Amplicons

**Figure 1:**
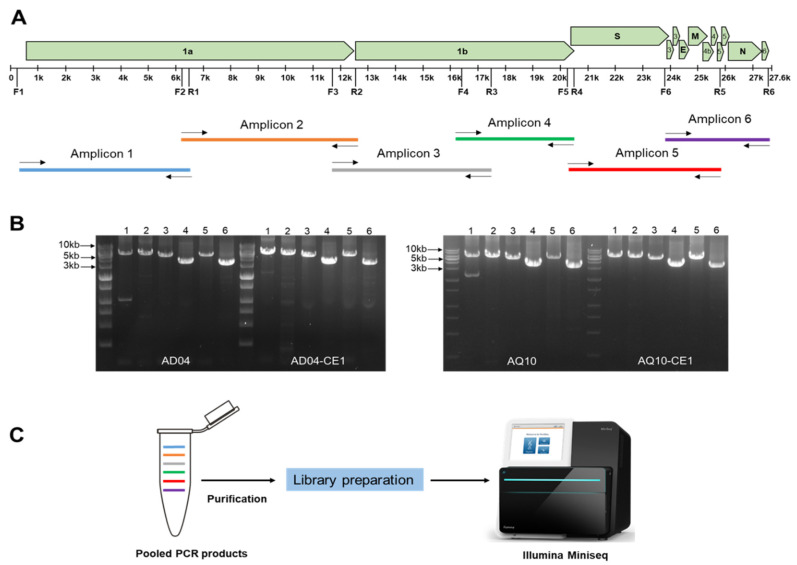
Overview of amplicon-based sequencing. (**A**) The locations of the six primer pairs on the IBV genome and their positions relative to those of the IBV genes. The regions amplified by each primer set and the overlap between the PCR products are also illustrated. (**B**) PCR results of the IBV genome amplification from the clinical and isolated samples. (**C**) PCR products were pooled in equal amounts, purified, and subjected to library preparation for Illumina sequencing.

The RT-qPCR results showed that all the samples were IBV positive. The range of Ct values of the four samples ranged from 19.1 to 23.17. The six primer sets were designed to target highly conserved regions of the IBV genomes (Table 1). The presence of amplicons of the expected size was confirmed using 1% agarose gel electrophoresis. Six overlapping fragments were successfully obtained via long PCR amplification from both the clinical and isolated samples (Figure 1). 

### 3.2. Complete Genome Characterization by Amplicon Sequencing

**Figure 2:**
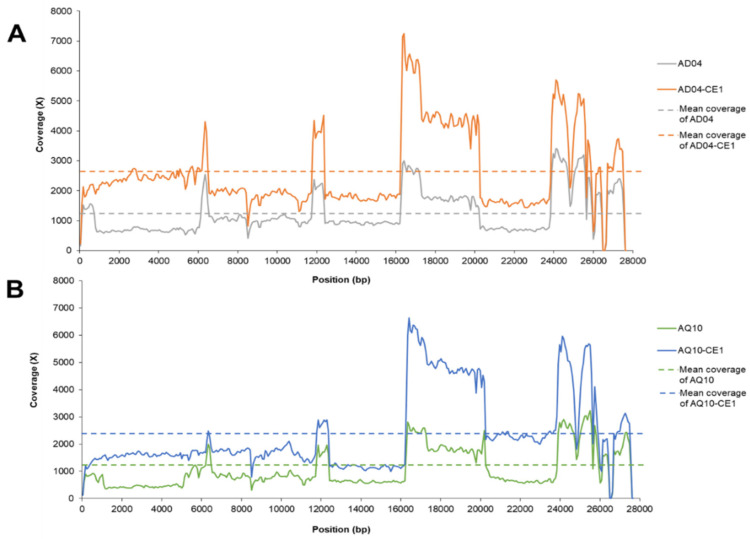
Coverage across the reference genome by the amplicon sequences from the AD04 and AD04-CE1 samples (**A**) and AQ10 and AQ10-CE1 samples (**B**). The *x*-axis represents the length of the reference genome YX10 (accession no. JX840411; 27,674 nucleotides). The *y*-axis represents the coverage (X).

The full-length IBV genomes of both the clinical samples and their respective isolates were successfully obtained using amplicon-based Illumina sequencing. The coverage rates of the IBV were 100% in all four amplicon sequencing samples. There were 13 ORFs (5′-UTR-1a-1ab-S-3a-3b-E-M-4b-4c-5a-5b-N-6-3′-UTR) identified within the IBV genomes. The mean depth of coverage of AD04 and AD04-CE1 was 1239 and 2629, respectively. Similarly, the AQ010 and AQ010-CE1 samples had mean coverage depths of 1069 and 2385, respectively. The coverage depth across the reference genome of the sequencing samples is shown in Figure 2.

**Figure 3:**
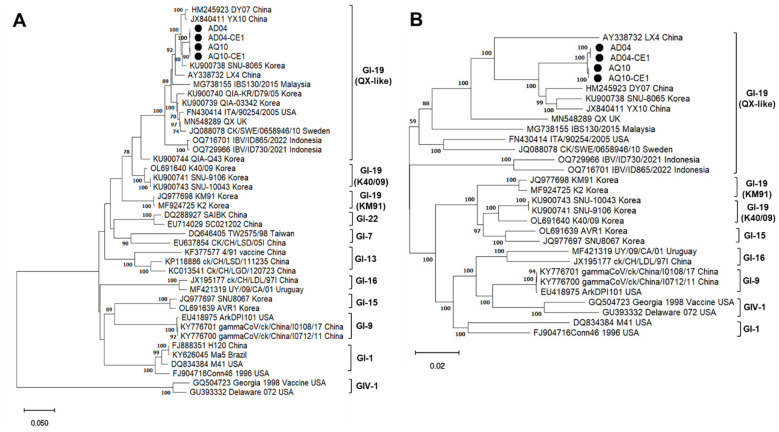
Phylogenetic analyses based on the S1 (**A**) and full-genome (**B**) sequences of AD04, AD04-CE1, AQ10, and AQ10-CE1 obtained by amplicon sequencing, and the reference IBV strains. The maximum likelihood method in Mega 11 software was used, with 1000 bootstrap replicates.

Phylogenetic analysis of the four amplicon sequencing samples and IBV reference strains was performed using the full-length S1 gene and full-length IBV genomes, respectively. The results showed that the sequences of AD04, AD04-CE1, AQ10, and AQ10-CE1 were of the GI-19 genotype (Figure 3).

The full-genome sequences of AD04 and AQ10 shared a 99.58% identity. The S1 genes of AD04 and AQ10 had high nucleotide and amino acid identities of 99.88% and 99.81%, respectively. The nucleotide and amino acid sequence identities of the S1 gene between the AD04 and 18 IBV isolates in the GI-19 branch of the phylogenetic tree (Figure 3) were 84.75–98.02% and 85–97.59%, respectively, while those between the AD10 and GI-19 IBVs were 84.69–98.02% and 85.19–97.78%, respectively.

### 3.3. Comparison of Amplicon Sequencing and Metagenome Sequencing Approaches for IBV

Next, we conducted a comparison between the results of the amplicon sequencing and metagenome sequencing. Among the four metagenome sequencing samples, the full-length sequence of IBV was successfully obtained from only the AD04-CE1 and AQ10-CE1 samples. All the isolated samples used for the sequencing showed 100% coverage rates for the IBV full genome. The sequences of AD04-CE1 and AQ10-CE1 obtained by metagenomic sequencing were annotated, with the IBV genome structure comprising 13 ORFs, similar to that of the amplicon-based sequencing samples. The mean coverage depths of AD04-CE1 and AQ010-CE1 were 864 and 4525, respectively. However, we were unsuccessful in acquiring the complete genome of IBV from the clinical samples using the SISPA approach. AD04 (with a Ct value of 22.3) showed a genome coverage of 22.58%, with a mean depth coverage of 14. The AQ10 (with a Ct value of 19.1) exhibited a genome coverage of 95.37%, with a mean depth coverage of 1148 (Table 2).

**Figure 4:**
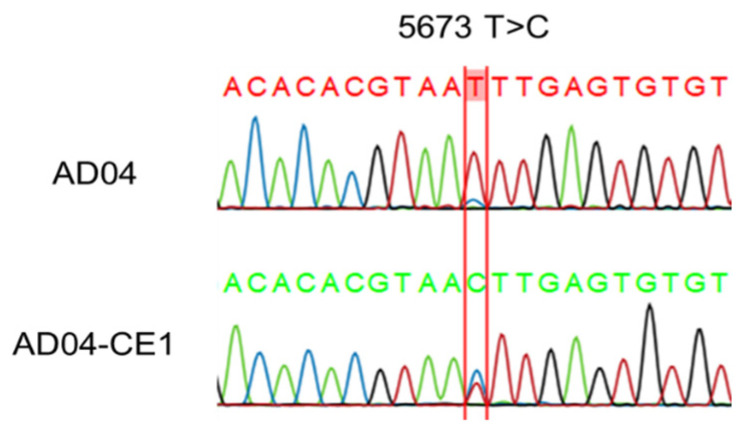
Sanger sequencing confirmation of a single nucleotide difference between the AD04 and AD04-CE1 samples.

A pairwise comparison of the IBV genome between AQ10 and AQ10-CE1 showed 100% similarity. However, there was a single nucleotide difference. Specific primer pairs were designed to confirm the substitution by Sanger sequencing. The Sanger sequencing results confirmed the presence of a single nucleotide substitution of T for C at position 5673 in the ORF1b between AD04 and AD04-CE1. However, this substitution did not lead to an amino acid change in the 1b polyprotein (Figure 4).

## 4. Discussion

Whole-genome sequencing, combined with various NGS protocols, is a powerful tool for investigating genome epidemiology and understanding the evolutionary dynamics of viruses [21,22,23,24,25]. Nonspecific amplification of purified nucleic acids in combination with mNGS allows all the genetic material directly recovered from a sample to be sequenced and analyzed, and SISPA represents one such metagenomic approach [23,24,25]. This method involves random priming and avoids the selection of specific RNA sequences, thus minimizing the biases associated with amplicon-based sequencing and allowing the detection of multiple pathogens [22,23]. However, in clinical samples, the low viral genetic loads and the overwhelming proportion of sequencing reads from the host DNA pose challenges to achieving the complete genome sequences of viruses by metagenomic sequencing [29,30,40,41]. In this study, the clinical samples, AD04 and AQ10, exhibited relatively low proportions of mapped reads to the reference genome, at 0.85% and 44.63%, respectively, demonstrating the limitation in obtaining the complete genome sequences of IBV directly from clinical samples using SISPA-mNGS.

To overcome these difficulties, the targeted enrichment of viral genomes from clinical samples using PCR has been used to reduce the nonspecific reads, streamline the bioinformatics analysis, and allow greater accuracy compared with metagenomics [31]. Recent studies have demonstrated that amplicon-based approaches offer greater genome coverage and higher sensitivity than mNGS [40,41,42,43]. These protocols are designed to amplify short and overlapping amplicons (about 1000 bp or less) across the genome and have been used to sequence the Zika virus, Crimean–Congo hemorrhagic fever virus, and SARS-CoV-2 from clinical samples [40,41,44,45]. However, they have not been successful in sequencing coronaviruses because these viruses have a high mutation rate that makes it difficult to design multiple primer pairs that do not result in primer mismatches [44,45]. 

In this study, we developed a protocol based on long-amplicon PCR to reduce the number of reactions. Six primer sets for amplicon sizes of 3.7–6.4 kb were designed to cover the whole IBV genome across various lineages, including GI-19. The primer sets successfully amplified bands of the correct size from two IBV strains, and sequencing of the amplicons using the Illumina MiniSeq platform resulted in high read accuracy and high levels of sequencing depth. These results indicate that the targeted long-amplicon PCR method provides high genomic coverage, with a mean depth of genome coverage of more than 1000-fold and high read mapping percentages ranging from 95.52% to 97.66%, in clinical and isolate samples.

Until now, mNGS for IBV whole-genome sequencing was usually performed on IBV isolates amplified by egg inoculation [26,27,28]. However, it is also important to conduct mNGS on IBV-infected clinical samples to detect co-infection with various IBV strains [46]. Moreover, there is a concern that viral passage through eggs or cell cultures can introduce single nucleotide substitutions, insertions, deletions, and recombination not initially present in the original clinical sample, potentially complicating the analysis [46,47]. The results of this study also revealed a single nucleotide difference between the genomes of IBV in the AD04 and AD04-CE1 samples, confirming that genetic variation during egg serial passage is a real possibility. This suggests that the introduction of a method to directly analyze the whole-genome sequence of the IBV from clinical samples using the long-amplicon-based approach would be useful for studying IBV mutations and evolution.

Among the IBVs, the QX-type subgroup belonging to the lineage GI-19 is widely prevalent in chicken flocks globally and currently predominant in South Korea [9,10,11]. In recent years, a variety of distinct QX-like IBVs (QX-I to IV) have been reported, as well as various recombinants [16,18]. Previous studies have demonstrated that recombination breakpoints were detected not only in the S1 gene but also in other parts of the IBV genome [15,16,17,18]. The construction of phylogenetic trees and sequence analysis suggested potential recombinant strains genetically distinct from others, and then the putative recombination events were identified using specialized software [16,17]. In this study, sequence comparisons of the S1 gene and phylogenetic analysis of both the S1 gene and the whole-genome of AD04 and AQ10 demonstrated that these strains belonged to the QX-type.

We found that the six primer pairs developed in this study are sufficient for the efficient amplification of viral DNA for whole-genome sequencing analysis of the GI-19 lineage. In the future, it is expected that this protocol will be applied to the genomes of other subgroups of the GI-19 lineage. Additionally, the protocol may be applicable to various IBV lineages, such as GI-1 (Mass type), GI-13 (4/91 type), and GVI-1 (TC07-2 type), since the primer sets were designed for the purpose of detecting various lineages by in silico analysis. Further evaluations with serial dilutions of viral RNA and clinical samples exhibiting higher Ct values will also be necessary to assess the sensitivity of the protocol.

In conclusion, we developed a novel method based on long-amplicon-based Illumina sequencing for high-accuracy whole-genome sequencing of the GI-19 lineage. The results indicate that this method can be applied for the direct sequencing of the full genomes of IBV in clinical samples, allowing accurate identification of IBV prevalence patterns. Also, it could facilitate metagenomic sequencing of an IBV vaccine and/or field strains in multiple co-infected samples. Additionally, the method is efficient in that it reduces the time and cost by enabling whole-genome sequencing without the need for a virus isolation procedure, and it can be used in a wide range of areas, such as diagnosis, study of virus characteristics, and effective vaccine programs on poultry farms. 

## Figures and Tables

**Table 1:** List of primers used to amplify six fragments of IBV.

Name	Primer Name	Sequence (5′ to 3′)	Position *	Product Size (bp)
Amplicon 1	IBF1	CTTAACAAAACGGACTTAAATACC	57	6429
	IBR1	GCAACYTCRGGAGACATAAATG	6485	
Amplicon 2	IBF2	GCAGGDTTYTATTTCTGGC	6226	6165
	IBR2	GTATCAGCCGAGCCTCACTG	12390	
Amplicon 3	IBF3	GAYCCACCATGTAARTTTGG	11749	5589
	IBR3	CRGGCTCRAAATTATTRCC	17337	
Amplicon 4	IBF4	GTATGTTRACCAAYTAYGAATTG	16264	3969
	IBR4	GTAAATARTTACWATTCCKCC	20232	
Amplicon 5	IBF5	GGTGGACAMTGTTYTGTACWG	20083	5719
	IBR5	CGMGCTTTTCKYGCTATTGC	25801	
Amplicon 6	IBF6	GACCTAARAARTCTGTTTAATG	23849	3721
	IBR6	CCCTCGATCGTACTCCGCG	27569	

* Nucleotide positions are relative to those of the YX10 strain (NCBI accession no. JX840411).

**Table 2:** Comparison between the amplicon-based sequencing and metagenome sequencing.

	Sample Name	Number of Read	Mapped Reads to Reference Sequence (%) ^a^	Coverage (%) ^b^	Mean of Depth Coverage ^c^	SD
**Amplicon-based sequencing**	**AD04**	271,693	265,341 (97.66%)	100	1239	711
	**AD04-CE1**	577,061	551,186 (95.52%)	100	2629	1313
	**AQ10**	258,588	249,866 (96.63%)	100	1069	714
	**AQ10-CE1**	482,990	470,782 (97.47%)	100	2385	1455
**Metagenome sequencing**	**AD04**	339,616	2,901 (0.85%)	22.58 ^d^	14	98
	**AD04-CE1**	578,112	193,793 (33.52%)	100	864	2170
	**AQ10**	552,618	246,618 (44.63%)	95.37 ^e^	1148	8406
	**AQ10-CE1**	1,218,715	989,833 (81.22%)	100	4525	24,717

^a^ Mapped reads to a reference sequence were calculated using the IBV reference strain YX10 (accession no. JX840411). ^b^ Coverage rate was calculated using the following formula: assembly contigs/reference genome. ^c^ Sequencing depth was calculated using the following formula: average read length × number of reads matching the reference/reference genome size. ^d^ Gaps at nucleotide positions: 1–32; 719–2387; 3486–7466; 7914–8436; 9398–11,769; 12,513–15,290; 16,312–20,627; 20,946–26,083; 27,061–27,674. ^e^ Gaps at nucleotide positions: 1–58; 16,933–17,152; 23,347–23,814; 24,106–24,617; 27,652–27,674.

## Data Availability

The sequences of the IBV strains in this study are available in the GenBank database under accession number PP374733 for AD04 and PP374734 for AQ10.

## Supplementary Materials

The following supporting information can be downloaded at https://www.mdpi.com/article/10.3390/v16040515/s1, Table S1: IBV reference strains used to design the primers in this study.
