# Supplementary material for: An Amplicon-Based Application for the Whole-Genome Sequencing of GI-19 Lineage Infectious Bronchitis Virus Directly from Clinical Samples
Source: Viruses. 2024 Mar 27;16(4):515. doi: 10.3390/v16040515 (PMC11054852; doi:10.3390/v16040515)
Supplement: Supplementary file 1 [file viruses-16-00515-s001.zip › viruses-2915134-supplementary.pdf]

viruses

MDPI

Supplementary

Table S1. IBV reference strains used to design the primers in this study.

No.	IBV strain	Genome length (nt)	Country	Accession number	Lineage (based on S1 gene classification)
1	M41	27476	USA	DQ834384	GI-1
2	H120	27652	China	FJ888351	GI-1
3	Ma5	27652	Brazil	KY626045	GI-1
4	Conn46 1996	27629	USA	FJ904716	GI-1
5	Holte	27247	USA	GU393336	GI-2
6	TW2575/98	27710	Taiwan	DQ646405	GI-7
7	CK/CH/LSD/05I	27638	China	EU637854	GI-7
8	gammaCoV/ck/China/I0108/17	27620	China	KY776701	GI-9
9	gammaCoV/ck/China/I0712/11	27623	China	KY776700	GI-9
10	ArkDPI101	27636	USA	EU418975	GI-9
11	AvCoV/Gallus gallus/Brazil/sample 38/2013	27618	Brazil	MG913342	GI-11
12	Brazil/SP55	27537	Brazil	MK953937	GI-11
13	D274	27599	Brazil	MH021175	GI-12
14	ck/CH/LSD/111235	27686	China	KP118886	GI-13
15	Ck/CH/LGD/120723	27718	China	KC013541	GI-13
16	4/91 vaccine	27618	China	KF377577	GI-13
17	B1648	27654	Belgium	KR231009	GI-14
18	NGA/A116E7/2006	27593	Nigeria	FN430415	GI-14
19	SNU8067	27708	South Korea	JQ977697	GI-15
20	AVR1	27679	South Korea	OL691639	GI-15
21	ck/CH/LDL/97I	27692	China	JX195177	GI-16
22	UY/09/CA/01	27647	Uruguay	MF421319	GI-16
23	IBV/Ck/Can/18-048192T	27638	Canada	MN512436	GI-17

24	Cal56b	27663	USA	GU393331	GI-17
25	ck/CH/AH/2020	27694	China	MW732689	GI-19
26	DK/CH/HN/ZZ2004	27673	China	JF705860	GI-19
27	BJ	27733	China	AY319651	GI-19
28	Partridge/GD/S14/2003	27503	China	AY646283	GI-19
29	DY07	27675	China	HM245923	GI-19
30	LX4	27528	China	AY338732	GI-19
31	YX10	27674	China	JX840411	GI-19
32	QX	27660	UK	MN548289	GI-19
33	CK/SWE/0658946/10	27664	Sweden	JQ088078	GI-19
34	ITA/90254/2005	27643	USA	FN430414	GI-19
35	21L068OP	27727	South Korea	OL691638	GI-19
36	KM91	27622	South Korea	JQ977698	GI-19
37	SNU-8065	27679	South Korea	KU900738	GI-19
38	QIA-03342	27684	South Korea	KU900739	GI-19
39	QIA-KR/D79/05	27682	South Korea	KU900740	GI-19
40	SNU-9106	27684	South Korea	KU900741	GI-19
41	SNU-10043	27683	South Korea	KU900743	GI-19
42	QIA-Q43	27675	South Korea	KU900744	GI-19
43	K2	27626	South Korea	MF924725	GI-19
44	K40/09	27820	South Korea	OL691640	GI-19
45	JP/Shimane/98	27643	Japan	LC716901	GI-19
46	IBV/ID730/2021	27718	Indonesia	OQ729966	GI-19
47	IBV/ID865/2022	27775	Indonesia	OQ716701	GI-19
48	IBS130/2015	27660	Malaysia	MG738155	GI-19
49	SAIBK	27534	China	DQ288927	GI-22
50	SC021202	27660	China	EU714029	GI-22
51	ck/CH/SCYB/140913	27647	China	KU356856	GI-23

52	gammaCoV/Ck/Poland/G052/2016	27595	Poland	KY047602	GI-23
53	Georgia 1998 Vaccine	27620	USA	GQ504723	GIV-1
54	Delaware 072	27591	USA	GU393332	GIV-1
55	KrD1515	27789	South Korea	OL691641	GVI-1
